# Magnetotransport in quantum cascade detectors: analyzing the current under illumination

**DOI:** 10.1186/1556-276X-6-206

**Published:** 2011-03-09

**Authors:** François-Régis Jasnot, Nicolas Péré-Laperne, Louis-Anne de Vaulchier, Yves Guldner, Francesca Carosella, Robson Ferreira, Amandine Buffaz, Laetitia Doyennette, Vincent Berger, Mathieu Carras, Xavier Marcadet

**Affiliations:** 1 Laboratoire Pierre Aigrain, Ecole Normale Supérieure CNRS (UMR 8551), 24 rue Lhomond, 75231 Paris Cedex 05, France; 2 Laboratoire Matériaux et Phénomènes Quantiques, Université Denis Diderot - Paris 7, CNRS (UMR 7162), Bâtiment Condorcet, 75205 Paris Cedex 13, France; 3 Alcatel-Thales 3-5 lab, Route départementale 128, 91767 Palaiseau Cedex, France

## Abstract

Photocurrent measurements have been performed on a quantum cascade detector structure under strong magnetic field applied parallel to the growth axis. The photocurrent shows oscillations as a function of *B*. In order to describe that behavior, we have developed a rate equation model. The interpretation of the experimental data supports the idea that an elastic scattering contribution plays a central role in the behavior of those structures. We present a calculation of electron lifetime versus magnetic field which suggests that impurities scattering in the active region is the limiting factor. These experiments lead to a better understanding of these complex structures and give key parameters to optimize them further.

## Introduction

The quantum cascade detector (QCD) [1] recently proposed and realized in both the mid-infrared [2] and the THz [3,4] range is a photovoltaic version of the quantum well infrared photodetector [5]. Their band structure are designed as quantum cascade lasers without any applied bias voltage [1,3]. QCD are totally passive systems and show a response only to photon excitation. As such, the QCD structure is designed to generate an electronic displacement under illumination through a cascade of quantum levels without the need of an applied bias voltage.

In a semiconductor quantum well structure, magnetic field applied along the growth direction breaks the 2D in-plane continuum into discrete Landau levels (LLs). This experimental technique has been used to evaluate the different contributions of scattering mechanism in complex quantum cascade structures [5-9].

We present in this article experimental photocurrent measurements under magnetic field applied along the growth direction. We develop a simple model of transport under illumination in a QCD. Through a comparison between experimental and calculation results, we evidence the mechanism limiting the response of the QCD.

## Experimental setup and sample

**Figure 1:**
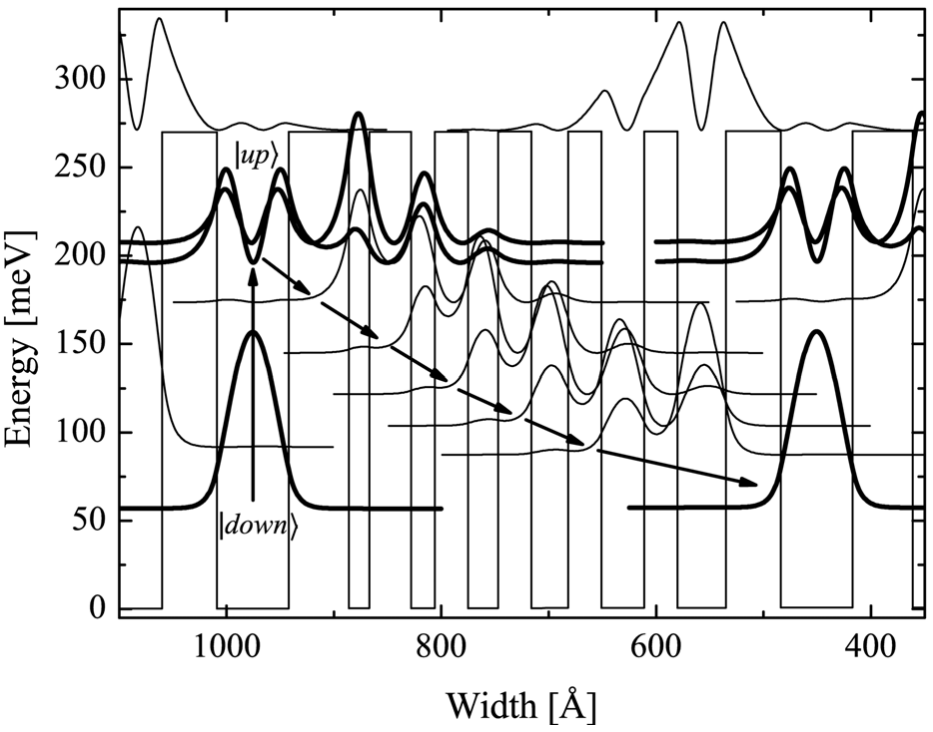
**Conduction band diagram of one period of an 8 *μ*m QCD showing the energy levels**. Note that the ground state of the first QW belongs to the former period and is noted |down〉. The arrows illustrate the electronic path during a detection event. The layer sequence is as follows 67.8/**56.5**/19.8/**39.6**/22.6/**31.1**/28.3/**31.1 **/33.9/**31.1**/39.6/**31.1**/45.2/**50.8 **(the barriers are represented in bold types). The *n*-doping of the large QW is 5 × 10^11 ^cm^-2^.

The QCD under study is a GaAs/Al_0.34_Ga_0.66_As heterostructure with a detection wavelength of 8 *μ*m as described in ref. [9]. It consists of 40 identical periods of 7 coupled GaAs quantum wells. Figure 1 recalls the principle of the device.

QCDs are mounted inside an insert at the center of a superconducting coil where a magnetic field *B *up to 16 T can be applied parallel to the growth axis. Light is emitted by a globar source from an FTIR spectrometer and guided to the sample. The experiment consists in measuring the current under illumination (*I*_light_) without any applied voltage at 80 K while the magnetic field is swept from 0 to 16 T.

## Results

**Figure 2:**
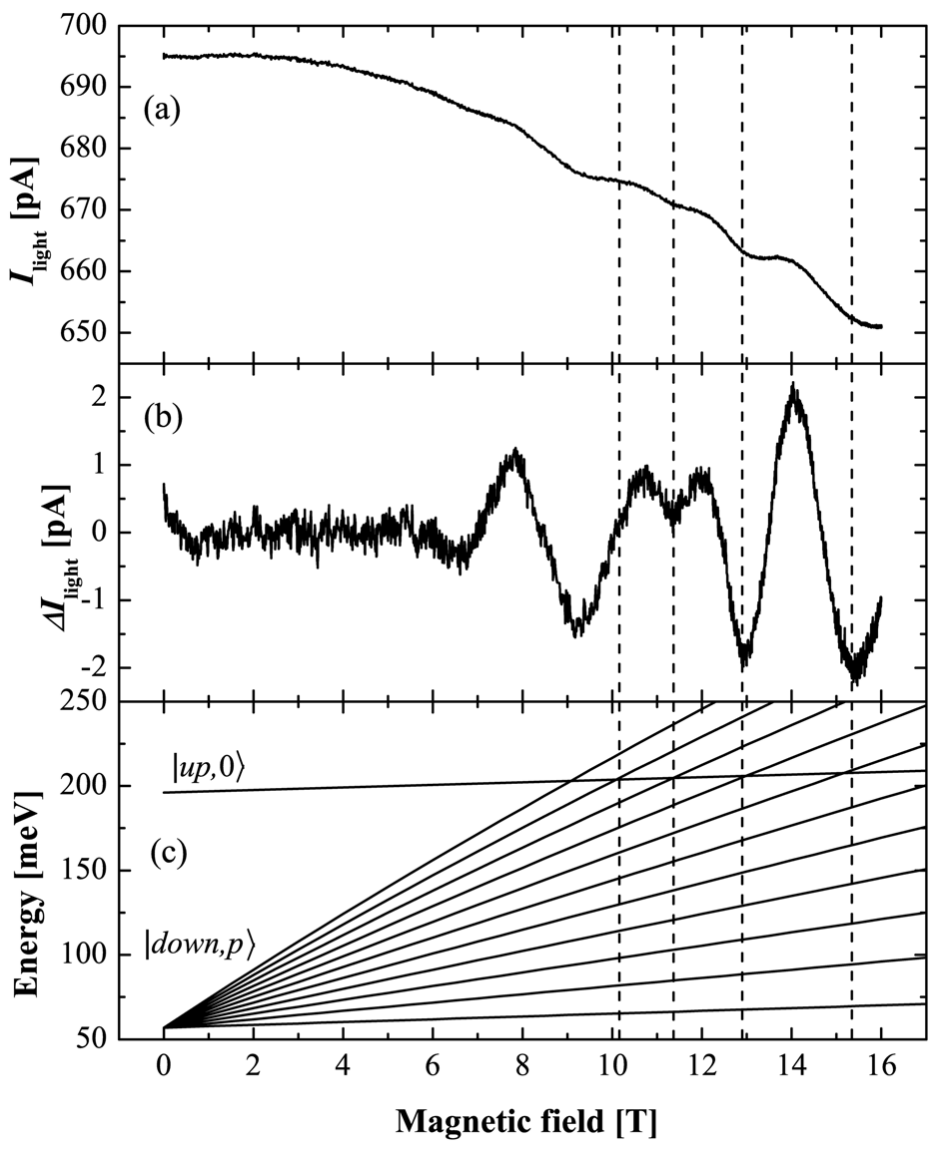
**Experimental result and LL fanchart**. **(a) **Current under illumination as a function of *B *at 80 K and at zero bias. **(b) ***I*_light _as a function of *B *where the decreasing background as been subtracted. **(c) **Fan chart of |up, 0〉 and |down, *p*〉 as a function of *B *taking into account the band non-parabolicity.

Experimental result is illustrated on Figure 2a. The photocurrent shows oscillations as a function of the magnetic field, superimposed on a continuous decreasing background which is removed from the experimental data in Figure 2b. Minima of current are located at *B *= 10.1, 11.4, 13.0, and 15.3 T and are in agreement with crossing of LL |up, 0〉 and LLs |down, *p*〉 represented on Figure 2c. It leads to the conclusion that an elastic scattering mechanism is dominant in this structure and mainly involves the levels |up〉 and |down〉.

## Discussion

We propose a model of transport in one period based on a rate equation approach. We assume that electrons are in the upper detector state |up〉 through absorption of a photon. Current as a function of lifetimes involved in this structure can be written:(1)

The parameters α and *N*_down _are, respectively, the absorption factor and sheet density of |down〉 and are constant. The subscribe *c *stands for the whole cascade. The quantum efficiency QE is the ratio of the lifetime τ_up-down _divided by τ_up-down _+ τ_up-c _and corresponds to the fraction of electrons on the level |up〉 that contributes to the photocurrent. In our model we suppose that any incident photon generates an absorption between the levels |down〉 and |up〉.

We present in Table 1 the calculated scattering rates of the different processes at *B *= 0 T. For interface roughness, we used a Gaussian autocorrelation of the roughness, with an average height of Δ= 2.8 Å and a correlation length of Δ = 60 Å. LO phonon emission scattering rate has been calculated as in ref. [10]. In our structure impurities scattering is the most efficient process [11]. Usually in GaAs quantum cascade structures this mechanism is neglected because the doped layers are not in the active region. In order to take into account the main scattering process we calculate ionized impurities scattering as a function of magnetic field. The details of the calculation are presented elsewhere [12].

**Table 1:** Calculated scattering rates in s^-1^.

Scattering mechanism	1/τ_up-down_	1/τ_up-c_
LO phonon emission	7.0 × 10^11^	7.2 × 10^11^
Interface roughness	6.0 × 10^11^	8.6 × 10^12^
Impurity scattering	1.8 × 10^13^	5.2 × 10^13^

Calculations are performed using different scattering processes for an electron in the |up〉 subband at *B *= 0 T.

**Figure 3:**
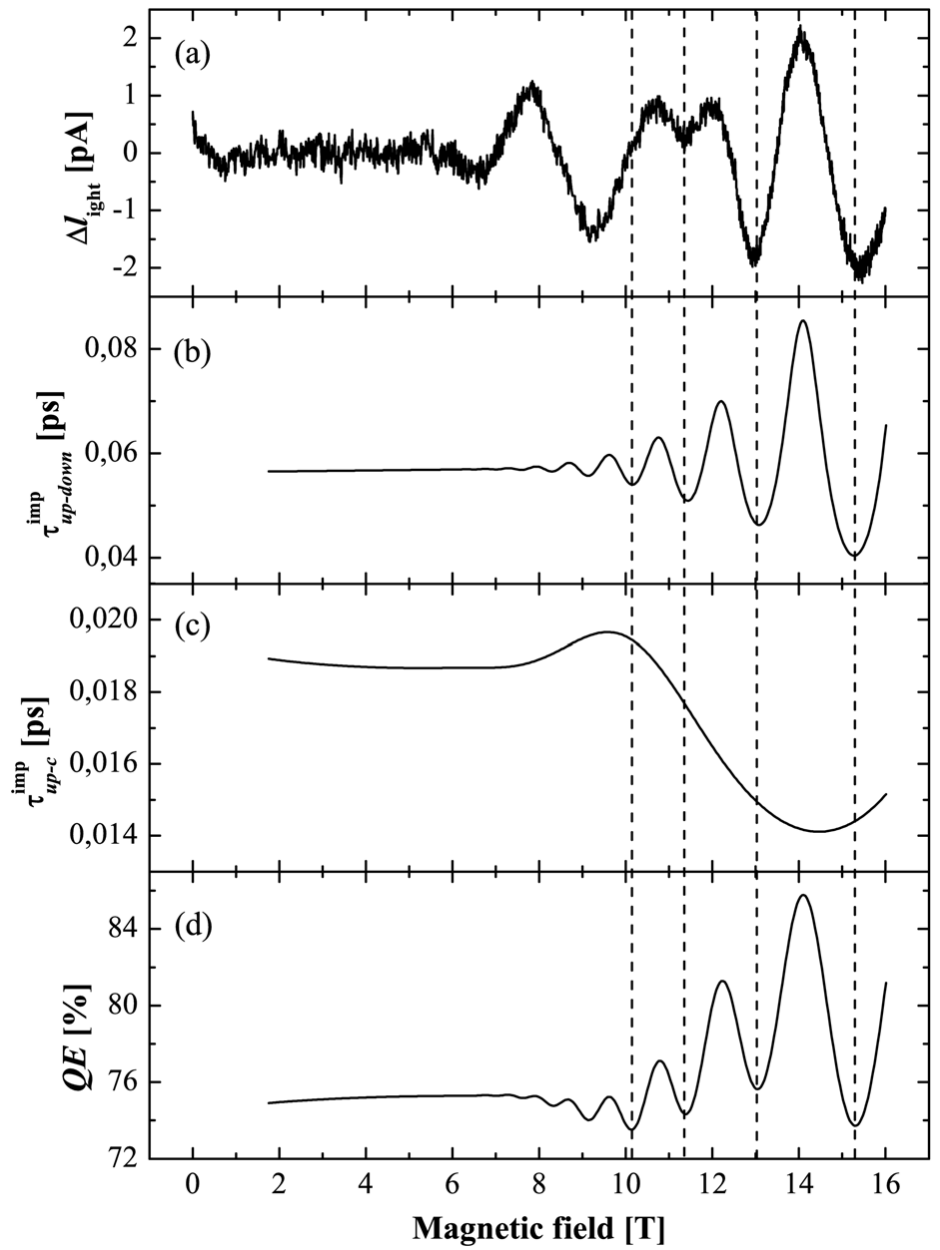
**Comparison between experimental data and electron-ionized impurities scattering time as a function of *B***. **(a) ***I*_light _as a function of the magnetic field where the background has been subtracted. **(b) **Ionized impurity scattering  under magnetic field between |up〉 and |down〉 levels. **(c) **Ionized impurity scattering  under magnetic field between |up〉 and levels in the cascade. **(d) **QE calculated with Equation (1).

Figure 3 represents a comparison between experimental data and electron-ionized impurities scattering time as a function of magnetic field. Figure 3b, c shows the two lifetimes involved in Equation 1 as a function of *B *calculated with electron-ionized impurities scattering. Figure 3d shows the calculation of the related quantum efficiency.

The oscillating behavior at high magnetic field (*B *> 9T) is a result of the electronic transfer from |up〉 to |down〉. This transfer leads to minima in the current which fit well with  and QE. The long period oscillating behavior of  as a function of *B *enhances the peak at *B *= 14 T in QE in agreement with experimental data. QE, which describes the performance of the detector, is oscillating between 74 and 85% under *B*. By extrapolating, at *B *= 0T, QE is equal to 75%, a value that should be increased to improve the detector performance. An optimized structure should take these results into account by shifting the ionized impurities from the active region, where they are enhancing , to a position where they would only enhance . The series of peak at *B *< 9T corresponds to a characteristic energy of 37 meV. This energy is attributed to transitions in the cascade involving an elastic scattering mechanism.

## Conclusion

In conclusion, we observe oscillations of the photocurrent in a mid-infrared QCD as a function of *B*. These oscillations are due to electron-ionized impurities scattering. This mechanism is dominant in this structure because impurities are located in the active region. In order to improve further this efficiency, we suggest to shift the impurities in another location of the structure in order to minimize .

The Laboratoire Pierre Aigrain is a " Unité Mixte de Recherche" between École Normale Supérieure, the CNRS, the University Paris 6 and the University Paris 7.

## Abbreviations

LLs: Landau levels; QCD: quantum cascade detector.

## Competing interests

The authors declare that they have no competing interests.

## Authors' contributions

FRJ, NPL and LAV performed magneto-transport experiment, analysed the data and drafted the manuscript. YG, FC and RF participated in the analysis of the data. AB, LD and VB designed the band diagram of the structure and performed analysis. MC and XM have grown the sample by molecular beam epitaxy. All authors read and approved the final manuscript.
